# Sex-Specific Variation in Maternal Serum Screening Markers Across the First and Second Trimesters: Evidence from 10,384 Screened Pregnancies

**DOI:** 10.3390/jcm15031276

**Published:** 2026-02-05

**Authors:** Mehmet Çopuroğlu, Hüseyin Aksoy, Mehmet Genco, Merve Genco, Cemal Ünlü

**Affiliations:** 1 Department of Obstetrics and Gynecology, Kayseri City Hospital, Kayseri 38030, Turkey; drhuseyinaksoy77@hotmail.com (H.A.); m.genco@hotmail.com (M.G.); 2 Department of Obstetrics and Gynecology, Kayseri State Hospital, Kayseri 38030, Turkey; mervedogan93@windowslive.com; 3 Department of Obstetrics and Gynecology, Special Clinic, Kayseri 38050, Turkey; cunlu06@gmail.com

**Keywords:** fetal sex, maternal serum markers, aneuploidy screening, first trimester, second trimester

## Abstract

**Background**: Maternal serum screening remains widely implemented for prenatal aneuploidy assessment despite increased uptake of cell-free DNA testing. Evidence suggests that fetal sex may influence placental endocrine function and maternal serum biomarker levels; however, available studies are inconsistent and often limited by sample size or incomplete adjustment for maternal factors. **Objective**: The aim of this study was to determine whether fetal sex independently modifies first- and second-trimester maternal serum marker Multiple of the Median (MoM) values and whether sex-related biochemical variation affects trisomy-21 screen-positive classification. **Methods**: A retrospective cohort was identified from institutional screening records (10,384 screened pregnancies), of which 5040 first-trimester and 1476 second-trimester cases had complete biochemical data. First-trimester PAPP-A and free β-hCG, as well as second-trimester AFP, uE3, and free β-hCG, were measured. Implausible MoM values (<0.10 or >5.00) were excluded. Multivariable linear and logistic regression models adjusted for maternal age, maternal weight, gestational age at sampling, and parity assessed independent associations. **Results**: Pregnancies with female fetuses showed significantly higher MoM values for first-trimester PAPP-A and free β-hCG as well as second-trimester AFP and uE3. The magnitude of these differences was small, and no significant differences were observed in trisomy-21 screen-positive rates between fetal sex groups. **Conclusions**: Fetal sex independently influences several maternal serum markers across both trimesters but does not result in clinically meaningful differences in trisomy-21 screening outcomes under current algorithms. Any potential relevance of fetal sex for risk interpretation should be regarded as hypothesis-generating and requires outcome-validated investigation before clinical application.

## 1. Introduction

Maternal serum screening in the first and second trimesters continues to constitute a fundamental component of prenatal aneuploidy assessment in many countries, even in the era of cell-free DNA-based testing. Although non-invasive prenatal testing (NIPT) has substantially improved detection performance for trisomy 21 and related conditions, conventional combined and triple tests remain widely implemented due to cost considerations, health-system accessibility, and established clinical pathways [1,2]. As a result, the clinical precision and calibration of maternal serum markers—particularly pregnancy-associated plasma protein A (PAPP-A), free β-human chorionic gonadotropin (free β-hCG), alpha-fetoprotein (AFP), unconjugated estriol (uE3), and inhibin-A—remain highly relevant to prenatal care in diverse populations.

Multiple maternal characteristics are known to influence serum marker distribution and MoM calculations, including gestational age, maternal age, body mass index, parity, and diabetes [3]. Despite this, fetal sex—although biologically plausible as a determinant of placental endocrine and metabolic function—has not been routinely incorporated into widely used risk algorithms. Evidence from developmental biology suggests sex-specific differences in placental vascularization, trophoblast function, and fetal endocrine signaling, implying that maternal serum markers could exhibit systematic variation according to fetal sex [4,5,6]. Given that MoM-based normalization represents a fundamental pre-analytic adjustment in screening algorithms, identifying biological determinants that systematically shift MoM distributions may have implications for population-specific calibration and risk interpretation.

Recent studies have reported modest sex-related differences in first-trimester free β-hCG and PAPP-A, as well as second-trimester AFP, uE3, and inhibin-A [7,8]. However, many of these investigations were limited by relatively small cohorts, single-trimester analyses, heterogeneous laboratory platforms, or restricted adjustment for maternal covariates. Moreover, available reports rarely assessed whether fetal sex alters calculated trisomy-21 risks or screen-positive rates after statistical control of key confounders. Consequently, the potential contribution of fetal sex as a meaningful modifier—beyond a purely biological observation—remains insufficiently characterized in contemporary prenatal screening practice.

In contrast to prior work, the present analysis is based on a large, single-center population comprising more than ten thousand pregnancies with standardized laboratory procedures, MoM normalization, and detailed maternal covariates. This scale enables precise quantification of sex-specific variation across both trimesters and robust evaluation of whether such differences extend beyond biomarker shifts to influence calculated risk and clinical screen-positive outcomes. Importantly, these data contribute sex-specific evidence from a middle-income setting, addressing a notable gap in a literature predominantly derived from Western populations. Rather than proposing a novel risk-calculation algorithm, this study aims to clarify whether fetal sex represents a systematic biological source of MoM variation that may warrant consideration in future calibration strategies. Clarifying whether fetal sex influences risk estimates near established screening thresholds may provide clinically relevant insight for refining population-specific screening performance. Accordingly, the objective of this study was to examine sex-related differences in first- and second-trimester maternal serum marker MoM values and to evaluate whether these differences translate into meaningful variation in trisomy-21 screen-positive classification under current screening algorithms.

## 2. Methods

### 2.1. Study Design and Setting

This study was designed as a retrospective analytical cohort and was conducted at a tertiary university-affiliated maternity center in Turkey. The institution serves as a regional referral center for prenatal care, where first- and second-trimester maternal serum screening is routinely offered as part of the national antenatal screening program. All biochemical screening tests and ultrasound examinations are performed according to standardized institutional protocols that follow national and international guidelines for aneuploidy screening. The study period extended from January 2019 to December 2022, during which all eligible pregnancies undergoing first- and/or second-trimester serum screening were identified from the institutional electronic screening registry.

Because the study used an existing database originally generated for clinical purposes, no changes were made to clinical management, and no additional procedures were introduced for research purposes. The design, setting, and time frame were chosen to ensure a sufficiently large and contemporary cohort, allowing a robust examination of sex-specific differences in serum screening markers under real-world conditions.

### 2.2. Participants and Eligibility Criteria

The source population consisted of all pregnant women who attended the center for routine prenatal aneuploidy screening within the study period. Eligible pregnancies were those with singleton gestations, documented first-trimester combined tests (double tests) and/or second-trimester triple tests, and available fetal sex information. Gestational age at the time of screening was determined primarily by first-trimester crown–rump length measurements and cross-checked with last menstrual period when available, in line with institutional ultrasound dating protocols.

Exclusion criteria were predefined to reduce bias related to extreme or unreliable data. Pregnancies were excluded if there was a multiple gestation, if fetal sex could not be confirmed from either ultrasound examinations after 18 gestational weeks or neonatal records, if a chromosomal abnormality, genetic syndrome, or major structural anomaly was diagnosed prenatally or postnatally, or if essential demographic data such as maternal age or gestational age at sampling were missing. Biochemically, pregnancies were excluded when any serum analyte result fell outside biologically plausible ranges, defined a priori as <0.1 or >5.0 multiple of the median (MoM) after initial verification. During the study period, a total of 10,384 singleton pregnancies underwent serum aneuploidy screening at the study center (7038 first-trimester combined tests and 3346 second-trimester triple tests). After excluding records with implausible MoM values (<0.10 or >5.00) or missing key covariates, the final analytical cohorts comprised 5040 first- trimester and 1476 second-trimester pregnancies.

### 2.3. Blood Sampling and Biochemical Analyses

Maternal venous blood samples were obtained by trained nurses or phlebotomists from an antecubital vein, typically between 08:00 and 11:00 a.m. Routine fasting was not mandated by the national screening protocol; however, women were advised to avoid heavy meals before sampling when feasible. For first-trimester screening, blood samples were collected between 11 + 0 and 13 + 6 gestational weeks. For second-trimester screening, sampling was performed between 15 + 0 and 18 + 6 gestational weeks. Blood was drawn into serum-separator tubes, gently inverted several times, and allowed to clot at room temperature for 20–30 min. Samples were then centrifuged at approximately 1500–2000× *g* for 10–15 min, and serum was separated and analyzed within the same working day. If immediate analysis could not be performed, serum aliquots were stored at 2–8 °C for up to 48 h in accordance with manufacturer recommendations.

All biochemical analyses were performed in the same institutional core laboratory using automated chemiluminescent immunoassay platforms (e.g., Roche Elecsys^®^ (Mannheim, Germany), Beckman Coulter Access^®^ (Brea, CA, USA), or equivalent, depending on the year and batch). Although different platforms were used over the study period, all assays were standardized through MoM normalization based on platform-specific medians, minimizing systematic inter-platform variability. In the first trimester, PAPP-A and free β-hCG were quantified using two-site sandwich immunoassays designed to detect intact analytes in maternal serum. In the second trimester, AFP, free β-hCG, and uE3 were routinely measured, and inhibin-A levels were assessed when the assay was available as part of the triple or quadruple panel. AFP assays targeted the glycoprotein primarily produced by the fetal liver; uE3 assays quantified the unconjugated estrogen fraction derived from placental conversion of fetal adrenal and hepatic precursors; and inhibin-A assays measured dimeric glycoprotein produced mainly by the placenta.

For all analytes, raw concentrations were initially expressed in units appropriate to each assay (e.g., IU/L for hCG, ng/mL for AFP, and nmol/L for uE3). The laboratory software then automatically converted these values into MoM, using gestational-age-specific median values provided by the manufacturer and adopted by the institution as population reference medians. MoM values were already adjusted for maternal weight at the time of calculation. Ethnicity-specific corrections were not applied because the study population was relatively homogeneous.

Internal quality control procedures were implemented on each run using low-, medium-, and high-level control materials supplied by the manufacturer. Intra-assay and inter-assay coefficients of variation remained within the ranges specified by the manufacturers (generally <7% and <10%, respectively). External quality assessment was conducted regularly through participation in national or international proficiency testing schemes, and the laboratory maintained continuous compliance with internationally accepted laboratory quality assurance and quality control procedures throughout the study period.

### 2.4. Variables and Outcomes

The main exposure of interest was fetal sex, categorized as male or female. Fetal sex was initially recorded from routine ultrasound examinations performed after 18 gestational weeks, where genital morphology allowed reliable determination, and was subsequently confirmed by neonatal records at birth. If discordant information was identified, the neonatal sex recorded in the delivery registry was considered definitive.

The primary outcome variables were the maternal serum marker MoM values obtained in the first- and second-trimester screening tests. Specifically, these included PAPP-A and free β-hCG in the first trimester, as well as AFP, free β-hCG, uE3, and inhibin-A in the second trimester. Because inhibin-A measurements were available only in a subset of second-trimester screenings due to assay availability, this marker was not included in the primary comparative and regression analyses.

Secondary outcomes included the calculated risk for trisomy 21 produced by the screening software and the proportion of pregnancies classified as screen-positive. Trisomy 21 risks were derived using the integrated laboratory software, which incorporates maternal age, gestational age, and the measured marker MoM values into a Bayesian risk algorithm based on Gaussian-distributed likelihood ratios. Risk calculations were performed using the institution’s routine screening software (PRISCA Prenatal Risk Calculation Software, version 5.2), which does not incorporate fetal sex as a parameter in baseline risk estimation. In accordance with national recommendations and institutional practice, a risk of 1:270 or higher for trisomy 21 was used to define a screen-positive result.

Additional recorded variables included maternal age, maternal weight, parity, smoking status when documented, and gestational age at the time of blood sampling. These variables were considered potential confounders and were therefore included in multivariable models.

### 2.5. Sample Size and Power Considerations

The study used an existing cohort defined by all eligible pregnancies within the specified time frame; thus, the final sample size of 10,384 pregnancies reflected the available data rather than a priori sample size planning. Nevertheless, to evaluate whether this sample offered adequate statistical power to detect clinically relevant sex-related differences in maternal serum markers, a post hoc power analysis was performed using G*Power 3.1 (Heinrich-Heine-Universität Düsseldorf, Düsseldorf, Germany). Assuming a two-sided independent-samples *t*-test, an alpha level of 0.05, and approximately equal group sizes for male and female fetuses, the available sample size provided >99% power to detect small effect sizes in marker MoM values (Cohen’s d ≈ 0.20). Even under more conservative assumptions of slightly unbalanced groups or smaller effect sizes, the power remained well above 90%.

For multivariable linear regression analyses, G*Power calculations assuming up to five covariates (maternal age, maternal weight, gestational age at sampling, parity, and smoking status) indicated that the available sample size was sufficient to detect very small incremental effects of fetal sex on marker MoM values (f^2^ ≈ 0.01) with a power exceeding 95% at α = 0.05. These considerations support that the study was adequately powered to identify modest, yet potentially clinically meaningful, sex-specific differences in maternal serum screening markers.

### 2.6. Statistical Analysis

All statistical analyses were conducted using IBM SPSS Statistics, version 25.0 (IBM Corp., Armonk, NY, USA). Continuous variables were assessed for distributional properties using histograms, Q–Q plots, and a Kolmogorov–Smirnov test. Normally distributed variables were summarized as mean ± standard deviation, whereas non-normally distributed variables were summarized as median with interquartile range. Categorical variables were presented as frequencies and percentages. Baseline maternal and pregnancy characteristics, as well as serum marker MoM values, were compared between male and female fetuses using independent-samples *t*-tests for normally distributed markers or Mann–Whitney U tests for skewed distributions. Chi-square tests were used for comparisons of categorical variables, such as screen-positive rates.

To examine the independent association between fetal sex and each serum marker, multivariable linear regression models were constructed with the marker MoM value as the dependent variable and fetal sex as the main independent variable. Models were adjusted for maternal age, maternal weight, gestational age at sampling, parity, and smoking status where available, based on their known or suspected influence on serum marker levels. Regression coefficients (β) and 95% confidence intervals were used to quantify the magnitude and precision of sex-related differences. To evaluate the relationship between fetal sex and the likelihood of a screen-positive result for trisomy 21, multivariable logistic regression models were fitted, adjusting for the same covariates. Adjusted odds ratios with 95% confidence intervals were reported.

In addition, exploratory interaction analyses were undertaken to assess whether the effect of fetal sex on serum markers varied across gestational age windows within the recommended screening intervals. Sensitivity analyses were performed after excluding pregnancies with maternal diabetes, those with extreme maternal weight (e.g., >120 kg), or those with missing smoking status, to examine the robustness of the findings.

Statistical significance was defined as a two-sided *p* value < 0.05 for all analyses, without formal adjustment for multiple comparisons due to the correlated nature of the biomarkers; however, effect sizes and consistency of findings across models were emphasized to avoid overinterpretation of statistically significant but clinically marginal differences.

### 2.7. Missing Data and Data Quality

Missing data and overall data quality were evaluated prior to analysis. Screening records initially included 10,384 singleton pregnancies, of which 5040 first-trimester and 1476 second-trimester cases had complete biochemical and demographic information available for analysis. Cases with missing fetal sex, incomplete screening marker values, or absent key covariates were excluded according to predefined criteria.

After applying these criteria, the proportion of excluded records due to missing or implausible MoM values (<0.10 or >5.00) remained low and did not exceed approximately 3% of the available datasets. Missingness was assumed to be predominantly missing completely at random (MCAR), given the administrative nature of exclusions and lack of clustering within specific subgroups; therefore, no imputation procedure was undertaken.

Outliers identified by MoM-based thresholds were cross-checked against original laboratory entries, where transcription inconsistencies were noted, values were corrected when possible, or the case was removed. This approach was chosen to ensure that the MoM-based standardization process accurately reflected biological rather than technical variation, which is critical for interpreting potential sex-specific shifts in marker distributions. All datasets ultimately analyzed were extracted directly from institutional electronic laboratory and screening systems, and data accuracy was confirmed by independent verification performed by two investigators.

### 2.8. Ethical Considerations

The study protocol was approved by the Institutional Ethics Committee of Kayseri City Hospital on 3 January 2023 (Approval no: 767). Because the study was based on retrospective analysis of anonymized clinical data, the requirement for individual informed consent was waived in accordance with local regulations and the policies of the ethics committee. All procedures were carried out in line with the principles of the Declaration of Helsinki and relevant national regulations on the protection of personal data.

## 3. Results

A total of 5040 first-trimester combined screening and 1476 second-trimester triple screening singleton pregnancies fulfilled all eligibility criteria and were included in the analytical cohorts after exclusion of missing key variables and implausible MoM values (<0.10 or >5.00).

Baseline maternal characteristics did not differ significantly by fetal sex in either cohort (all *p* > 0.05). In the first trimester, mean maternal age was 27.2 ± 5.6 years in male and 27.2 ± 5.7 years in female fetuses, with similar maternal weight (77.5 ± 13.5 vs. 77.3 ± 13.2 kg) and gestational age at sampling (12.5 ± 0.8 vs. 12.5 ± 0.8 weeks). In the second trimester, maternal age (26.8 ± 5.9 vs. 26.7 ± 5.7 years), weight (76.8 ± 13.6 vs. 76.6 ± 13.5 kg) and gestational age at sampling (17.3 ± 1.3 vs. 17.2 ± 1.2 weeks) were likewise similar for male and female fetuses. Median parity was 1 in all groups (Table 1).

After exclusion of implausible MoM values, analyzable data were available for 5022 pregnancies for PAPP-A and 4952 pregnancies for free β-hCG in the first-trimester cohort. Unadjusted analyses showed that female fetuses had higher mean MoM values for both PAPP-A and free β-hCG compared with male fetuses. Mean PAPP-A MoM was 1.05 ± 0.62 in females versus 1.01 ± 0.60 in males (*p* = 0.014). Mean free β-hCG MoM was 1.12 ± 0.56 in females versus 1.02 ± 0.52 in males (*p* < 0.001). Although statistically significant, the absolute between-sex differences in MoM values were small in magnitude. Female fetuses again showed slightly higher unadjusted mean AFP and uE3 MoM than male fetuses, whereas free β-hCG MoM was similar between groups. Mean AFP MoM was 1.17 ± 0.53 in female versus 1.12 ± 0.42 in male fetuses (*p* = 0.038). Mean uE3 MoM was 1.14 ± 0.35 in females versus 1.10 ± 0.32 in males (*p* = 0.009). Free β-hCG MoM values were 1.12 ± 0.62 in females and 1.10 ± 0.66 in males (*p* = 0.544) (Table 2).

In multivariable linear regression adjusting for maternal age, maternal weight, gestational age at sampling and parity, female fetal sex remained an independent predictor of higher PAPP-A and free β-hCG MoM values. Female sex was associated with a +0.043 MoM increase in PAPP-A (95% CI 0.011–0.075; *p* = 0.009) and a +0.100 MoM increase in free β-hCG (95% CI 0.070–0.129; *p* < 0.001) compared with male fetuses. These effect sizes correspond to small standardized differences, indicating modest biological shifts rather than large deviations in marker distributions. In adjusted models controlling for maternal age, weight, gestational age at sampling and parity, female sex remained significantly associated with higher AFP and uE3 MoM, but not with free β-hCG MoM. Female fetuses had a +0.053 MoM increase in AFP (95% CI 0.004–0.102; *p* = 0.032) and a +0.041 MoM increase in uE3 (95% CI 0.006–0.076; *p* = 0.020). The adjusted association between female sex and second-trimester free β-hCG MoM was small and not statistically significant (β = +0.035; 95% CI −0.030 to 0.100; *p* = 0.295) (Table 3).

In multivariable logistic regression analyses adjusted for maternal age, weight, gestational age at sampling and parity, female fetal sex was not significantly associated with the odds of a screen-positive result in either trimester. For first-trimester combined screening, the adjusted odds ratio (OR) for a screen-positive result in female versus male fetuses was 0.71 (95% CI 0.44–1.13; *p* = 0.152). For second-trimester triple screening, the adjusted OR was 0.96 (95% CI 0.52–1.76; *p* = 0.885) (Table 4). Thus, despite statistically detectable differences in individual marker MoM values, no meaningful variation in trisomy-21 screen-positive classification was observed.

## 4. Discussion

The present large-scale single-center analysis demonstrates that fetal sex exerts a statistically significant yet biologically modest influence on multiple maternal serum screening biomarkers across both first and second trimesters. Pregnancies with female fetuses exhibited higher MoM values for PAPP-A and free β-hCG during first-trimester screening, as well as higher AFP and uE3 during second-trimester screening. Importantly, these differences represent small shifts in marker distributions rather than large deviations at the individual-patient level. These findings align with the emerging notion that sexual dimorphism in placental function influences maternal serum biomarker profiles in early pregnancy [7,9].

Previous literature addressing fetal sex differences in first-trimester markers has been somewhat inconsistent. Early studies reported higher free β-hCG in female fetuses, but many suggested minimal or no difference in PAPP-A [10,11]. Subsequent larger datasets, however, indicated sex-specific elevation in both free β-hCG and PAPP-A [12,13]. From this perspective, the current results reinforce the more recent view that female fetuses are associated with a modest upward shift in first-trimester biomarker MoM levels. Importantly, unlike many earlier studies, the present analysis systematically adjusted for gestational age, maternal age, maternal weight and parity, thereby strengthening causal interpretation and minimizing residual confounding [14,15,16].

Evidence regarding second-trimester biomarkers has been relatively limited, fragmented and often based on small sample sizes. While selected reports noted higher AFP or inhibin-A in female fetuses, findings have not been consistent and were rarely evaluated in large contemporary cohorts [17]. The current results contribute novel evidence by demonstrating reproducible, statistically independent sex-related differences in second-trimester AFP and uE3, even after multivariable adjustment. These findings extend existing knowledge by suggesting that sex-dependent biomarker variation is not restricted to early gestation but persists into mid-pregnancy. Such variation likely reflects differences in placental metabolism and fetal hepatic or adrenal pathways [18,19].

The biological plausibility for these findings is supported by experimental and clinical studies describing sexual dimorphism in placental endocrine regulation, trophoblast invasion, vascular architecture and oxidative stress responses [20]. A growing body of research has demonstrated that male and female placentas differ in gene expression, hormonal profiles and susceptibility to adverse intrauterine conditions, implying that maternal serum biomarkers—whose origins include placental and fetal metabolic processes—might reflect these sex-specific patterns [21]. Within this biological framework, the observed MoM differences are coherent and unlikely to represent random analytical variation.

Clinically, one important question is whether sex-associated shifts in biomarker MoM values influence aneuploidy risk calculation or screen-positive classification. The present findings indicate that, although statistically significant differences exist in several MoM values, these changes did not translate into clinically meaningful differences in trisomy-21 screen-positive rates using a 1:270 cut-off. This suggests that current screening algorithms are generally robust to modest sex-related biomarker variation under routine clinical conditions.

Nevertheless, the consistent direction of sex-specific differences across multiple biomarkers raises the possibility that in specific scenarios—such as pregnancies with risk estimates near established cut-offs—small cumulative shifts could theoretically influence risk categorization. Importantly, this interpretation remains hypothetical, as no outcome-validated or borderline-risk subgroup analysis was performed in the present study. Accordingly, any potential clinical implications should be interpreted cautiously and viewed as a hypothesis-generating observation rather than evidence supporting immediate modification of screening algorithms [22,23].

A notable strength of this study is the very large sample size spanning two routine screening windows, enabling high statistical precision and robust multivariable modeling. Single-center design ensured consistent laboratory methodology, uniform MoM conversion and stable analytic conditions, thereby minimizing heterogeneity—a major limitation in prior multi-center reports. Furthermore, exclusion of biologically implausible MoM values and systematic adjustment for key maternal factors improve internal validity and reduce the likelihood that observed effects represent measurement artifact rather than physiological signal. The parallel evaluation of biomarker-level differences and screen-positive outcomes further supports a balanced interpretation of statistical and clinical relevance.

Several limitations merit consideration. First, the retrospective design precludes definitive causal inference and may be subject to residual confounding from unmeasured maternal factors, such as smoking status or subtle placental pathology. Second, because the study population was derived from a single country with limited ethnic variation, generalizability to more diverse populations should be interpreted cautiously. Third, although marker differences were statistically robust, they were small in magnitude; thus, the clinical implications, particularly regarding sensitivity and specificity for aneuploidy detection, require further investigation using outcome-confirmed datasets. Finally, given that chromosomal outcome data were not universally available, direct assessment of diagnostic performance was not possible.

Future research should therefore prioritize outcome-validated analyses and explore whether sex-specific reference ranges or MoM adjustments offer incremental benefit in defined clinical contexts. Prospective multicenter studies incorporating diverse populations, confirmed aneuploidy outcomes, and decision-impact analyses will be essential to determine whether fetal sex should be systematically integrated into prenatal screening algorithms. Until such evidence is available, the present findings should be interpreted primarily as biologically informative rather than practice-changing.

Taken together, these findings provide contemporary, population-based evidence that fetal sex systematically influences maternal serum screening markers in both first and second trimesters. While this biological variation does not appear to alter overall screening outcomes under current protocols, it highlights an underrecognized source of variability in prenatal screening biology. Recognition of sex-specific biomarker patterns may inform future refinement of screening models, particularly in population-specific or research settings, but does not currently justify routine sex-based adjustment in clinical practice.

## 5. Conclusions

This large, single-center cohort provides robust evidence that fetal sex exerts a statistically independent, modest influence on multiple maternal serum biomarkers routinely used in first- and second-trimester aneuploidy screening. While female fetuses demonstrated consistently higher MoM values for PAPP-A and free β-hCG in early pregnancy, as well as elevated AFP and uE3 in mid-gestation, these sex-related biochemical differences did not translate into clinically meaningful variation in calculated trisomy-21 risk or screen-positive classification under current algorithms.

The findings of this study support the biological plausibility of sex-specific placental and fetal endocrine pathways while indicating that routine sex-based adjustment of maternal serum screening is not required in general populations. The consistent direction of effect across biomarkers nevertheless highlights an underrecognized source of biological variability in prenatal screening markers.

Any potential relevance of fetal sex in borderline-risk scenarios should be interpreted cautiously and considered hypothesis-generating, as the present study did not include outcome-validated or subgroup-specific analyses. Accordingly, clinical implementation or algorithm modification cannot be recommended on the basis of these findings alone. Future prospective studies incorporating confirmed fetal outcomes and decision-impact analyses will be essential to determine whether fetal sex merits integration into population-specific screening models.

## Figures and Tables

**Table 1:** Baseline maternal and pregnancy characteristics by fetal sex (first- and second-trimester screening cohorts).

Cohort	Fetal Sex	*n*	Maternal Age, Mean ± SD (Years)	Maternal Weight, Mean ± SD (kg)	GA at Sampling, Mean ± SD (Weeks)	Parity, Median
**First-trimester combined test**	Male	2561	27.2 ± 5.6	77.5 ± 13.5	12.5 ± 0.8	1
**First-trimester combined test**	Female	2479	27.2 ± 5.7	77.3 ± 13.2	12.5 ± 0.8	1
**Second-trimester triple test**	Male	734	26.8 ± 5.9	76.8 ± 13.6	17.3 ± 1.3	1
**Second-trimester triple test**	Female	742	26.7 ± 5.7	76.6 ± 13.5	17.2 ± 1.2	1

**Table 2:** Unadjusted maternal serum marker MoM values by fetal sex (first and second trimesters).

**First Trimester**			
**Marker**	**Male, mean ± SD (MoM)**	**Female, mean ± SD (MoM)**	***p***
**First-trimester PAPP-A**	1.01 ± 0.60	1.05 ± 0.62	0.014
**First-trimester free β-hCG**	1.02 ± 0.52	1.12 ± 0.56	<0.001
**Second Trimester**			
**Marker**	**Male, mean ± SD (MoM)**	**Female, mean ± SD (MoM)**	***p***
**Second-trimester AFP**	1.12 ± 0.42	1.17 ± 0.53	0.038
**Second-trimester uE3**	1.10 ± 0.32	1.14 ± 0.35	0.009
**Second-trimester free β-hCG**	1.10 ± 0.66	1.12 ± 0.62	0.544

All MoM (Multiple of the Median) values were calculated using gestational-age-specific, weight-adjusted medians provided by the screening software.

**Table 3:** Adjusted association between female fetal sex and maternal serum marker MoM values (multivariable linear regression).

Marker	Beta for Female Sex (MoM)	95% CI	*p*
**First-trimester PAPP-A MoM**	+0.043	0.011 to 0.075	0.009
**First-trimester free β-hCG MoM**	+0.100	0.070 to 0.129	<0.001
**Second-trimester AFP MoM**	+0.053	0.004 to 0.102	0.032
**Second-trimester uE3 MoM**	+0.041	0.006 to 0.076	0.020
**Second-trimester free β-hCG MoM**	+0.035	−0.030 to 0.100	0.295

Multivariable linear regression adjusted for maternal age, maternal weight, gestational age at sampling and parity.

**Table 4:** Adjusted odds ratios for screen-positive trisomy 21 result (female vs. male fetus).

Outcome	Adjusted OR for Female vs. Male	95% CI	*p*
**First-trimester screen-positive**	0.71	0.44–1.13	0.152
**Second-trimester screen-positive**	0.96	0.52–1.76	0.885

Multivariable logistic regression adjusted for maternal age, maternal weight, gestational age at sampling and parity.

## Data Availability

The datasets analyzed during the current study are derived from institutional patient records and are not publicly available due to privacy and ethical restrictions. De-identified data may be made available from the corresponding author upon reasonable request and with institutional approval.
